# Functional Preservation and Reorganization of Brain during Motor Imagery in Patients with Incomplete Spinal Cord Injury: A Pilot fMRI Study

**DOI:** 10.3389/fnhum.2016.00046

**Published:** 2016-02-15

**Authors:** Xin Chen, Lu Wan, Wen Qin, Weimin Zheng, Zhigang Qi, Nan Chen, Kuncheng Li

**Affiliations:** ^1^Department of Radiology, Xuanwu Hospital, Capital Medical UniversityBeijing, China; ^2^Beijing Key Laboratory of Magnetic Resonance Imaging and Brain InformaticsBeijing, China; ^3^Department of Radiology, Tianjin Medical University General HospitalTianjin, China

**Keywords:** spinal cord injury, motor imagery, motor execution, functional magnetic resonance imaging, functional reorganization

## Abstract

Motor imagery (MI) is a cognitive process involved in mentally rehearsing movement representations, and it has great potential for the rehabilitation of motor function in patients with spinal cord injuries. The aim of this study was to explore changes in the brain activation patterns in incomplete spinal cord injury (ISCI) patients during motor execution (ME) and MI tasks, and to thereby explore whether MI shares similar motor-related networks with ME in ISCI patients. Seventeen right-handed ISCI patients with impaired motor function of their right ankles and 17 age- and gender-matched healthy controls were enrolled in this study. The activation patterns of the ISCI subjects and those of the healthy subjects were compared, both during mental dorsi-plantar flexion of the right ankle (the MI task) and the actual movement of the joint (the ME task). The patients and the healthy controls shared similar activation patterns during the MI or ME tasks. The activation patterns of the MI task between the patients and the healthy controls were more similar than those of the ME task. These findings indicate that the MI network is more functionally preserved than the ME network in ISCI patients. In addition, increased activation in the motor-related regions during ME task, and decreased activation in the parietal regions during both ME and MI tasks, were identified in the ISCI patients compared to the healthy controls, indicating a functional reorganization of these regions after ISCI. The functional preservation and reorganization of the MI network in the ISCI patients suggests a potential role for MI training in motor rehabilitation.

## Introduction

Motor imagery (MI) is defined as a dynamic state in which a subject mentally performs a specific motor action without actually producing a motor output (Guillot et al., 2012; Di Rienzo et al., 2014b). MI and motor execution (ME) share similar functional processes (Decety, 1996; Dickstein et al., 2014). Recently, MI has been used for improving the motor rehabilitation in stroke (Dickstein et al., 2014), Parkinson's syndrome (Wondrusch and Schuster-Amft, 2013), and complete spinal cord injury (CSCI) patients (Grangeon et al., 2012; Di Rienzo et al., 2014a).

Spinal cord injury disrupts the afferent somatosensory pathway and/or the efferent motor pathway. Several studies have demonstrated the potential role of MI in motor recovery (Cramer et al., 2007; Grangeon et al., 2012). Previous functional MRI studies (Alkadhi et al., 2005; Cramer et al., 2005, 2007; Hotz-Boendermaker et al., 2008; Gustin et al., 2010) have found that the brain activation patterns of the MI task in CSCI patients were largely similar to those of the attempted movements. Since the CSCI patients have completely lost their motor ability in the affected limbs, comparing the activation patterns between the MI and ME in the incomplete SCI (ISCI) patients (whose afferent and efferent pathways are partially preserved) may provide additional information about the relationship between MI and ME.

However, few neuroimaging studies of MI have focused on incomplete ISCI patients. ISCI is the most prevalent type of SCI, and it has a greater potential for motor recovery. It has been reported that earlier motor rehabilitation strategies can dramatically improve the outcome of ISCI (Sumida et al., 2001; Dietz and Fouad, 2014). If ISCI patients have preserved MI capability, it may have a valuable effect when used by ISCI patients for motor rehabilitation in the short period of the time after injury or surgery, during which the patients are not able to carry out effective ME rehabilitation. Moreover, ME training is more heavily dependent on the external conditions (e.g., the patients' economic condition, physical capacity, rehabilitation equipment and strategies) compared to MI. Thus, MI studies for the treatment of ISCI may have more clinical benefits to a wider population of patients. In order to design a MI rehabilitation protocol specifically for ISCI patients, it is necessary to explore the functional organization patterns of MI and the subsequent changes in this system in the ISCI patients.

In this study, we focused on the similarities and differences in the activation patterns of the MI and ME tasks in the ISCI subjects. Since rehabilitative training is known to induce structural brain reorganization in ISCI patients (Villiger et al., 2015), we chose patients without any specific rehabilitative training. Because previous studies had reported relatively preserved MI activation networks in the CSCI (Hotz-Boendermaker et al., 2008), and the motor function of the patients recruited in present study were not fully deprived, we hypothesized that both the MI and ME activation patterns of the ISCI subjects were similar to those of healthy subjects.

## Materials and methods

### Subjects

Seventeen right-handed patients with ISCI (10 male and 7 female patients, with a mean age of 38.3 ± 12.2 years, and a range of 21–55 years) were enrolled in this study. When manual muscle testing was performed on these cases, it was found that there was dorsiflexion impairment in the right ankle of all cases. Six patients were labeled grade C, while 11 were labeled grade D, according to the American Spinal Injury Association Impairment Scale 2012 (www.asia-spinalinjury.org). The course of the disease was 1.36 ± 1.62 years. We excluded the following patients from this study: patients with brain diseases that were confirmed by conventional MRI, patients who had suffered orthopedic trauma in their lower limbs, patients with a history of rehabilitation, and patients with any kind of mental illness or cognitive disorder. Table 1 provides detailed information on the ISCI patients enrolled in this study. Seventeen age- and gender-matched right-handed healthy volunteers (10 male and 7 female patients, with a mean age of 38.2 ± 12.3 years and a range of 21–57 years) were recruited to form the normal control (NC) group.

**Table 1:** **Clinical data for patients with ISCI**.

**Subject**	**Age (years)**	**Gender**	**Etiology**	**Duration (years)**	**Injury level**	**MRCS_MS**	**Pathologic reflex**	**ASIAIS**
1	50	M	Trauma	0.50	C5-7	3-	Y	C
2	37	M	Disc prolapse	3	C4-7	4	Y	D
3^*^	22	M	Spinal tuberculosis	0.50	C7-T3	3-	N	C
4	35	M	Disc prolapse	1	C2-5	4	N	D
5	21	M	Meningeal sarcoma	0.08	C4-5	4	Y	D
6	54	F	Neurilemmoma	1	C2-3	3	Y	D
7	24	F	Disc prolapse	2	C3-4	4	Y	D
8^*^	33	M	Trauma	0.04	L1	3-	N	C
9^*^	55	F	Disc prolapse	6	T6-7, T12-L1	3	Y	D
10	53	M	Disc prolapse	4	C3-7	4	Y	D
11	24	F	Neurilemmoma	2	C3-7	4	Y	D
12	38	F	Spinal hemorrhage	0.08	C2-6	3	N	D
13	49	M	Astrocytoma	1	C2-7	3	Y	D
14^*^	36	M	Neurilemmoma	0.75	C6-T2	3-	N	C
15	54	F	Trauma, disc prolapse	0.75	C3-4	4	N	D
16^*^	28	F	Trauma	0.58	T11	3-	N	C
17^*^	38	M	Trauma	0.08	T4	3-	Y	C

*The MRCS_MS and Pathologic reflex are measured on the right lower limb*.

* *Represents patients with sub-cervical spinal cord injuries. ASIAIS, American spinal injury association impairment scale. ISCI, incomplete spinal cord injury; MRCS_MS, medical research council scale for muscle strength*.

This study protocol was approved by the Ethics Committee of Xuanwu hospital, Capital Medical University, Beijing, China. Written informed consent in accordance with the Declaration of Helsinki was obtained from each participant of this study.

### Image acquisition

All participants were scanned on a 3.0 T whole-body Magneto Trio Tim MRI scanner (Siemens Healthcare, Forchheim, Germany) using a 12-channel receive head coil. fMRI experiments were carried out using a single-shot gradient echo-planar imaging sequence with the following parameters: repetition time/echo time= 2000/30 ms; flip angle = 90°; field of view = 220 × 220 mm^2^; matrix = 64 × 64; readout bandwidth, 2298 Hz/pixel; slice thickness = 3 mm; inter-slice gap = 1 mm; slices = 35. An intergrade parallel imaging technique with an acceleration factor of 2 was used to reduce the susceptibility-induced image distortion and signal loss. Tight but comfortable foam padding was used to minimize the head motion, and earplugs were used to reduce the imaging noise.

### Motor imagery and motor execution tasks

We chose a kinesthetic MI task (imagining oneself performing the given movements without the actual motor output) rather than a visual MI task (watching another person performing the movement) because kinesthetic MI is more functionally equivalent to ME (Lacourse et al., 2005). The ankle dorsi-plantar flexion (ADF) task was chosen as the motor task. ADF is an important component of the gait cycle, which is used to determine whether patients with extremely impaired lower motor function can recover their walking ability after suffering a SCI. Previous studies have shown that ADF has similar brain activation patterns to that of walking (Dobkin et al., 2004). The ADF restoration is a key element in ISCI patients to improve their walking capabilities (Varoqui et al., 2014). Hence, we used it as our fMRI paradigm.

The experimental design included 2 sequences (ME and MI). The ME sequence had 4 repetitions, which were alternated by a 20 s task block and a 20 s rest block (beginning with a task block and ending with a rest block) and so did the MI sequence. All participants were instructed to lie down in the supine position and relax with their eyes closed to avoid visual input. They were asked to keep still during the scan and to stop mentally rehearsing their movement during the rest blocks. During the ME task, the healthy controls were instructed to perform the ADF (20° dorsiflexion to neutral to 30° plantarflexion) at a self-paced rhythm of approximately 0.5–1.0 Hz. The ISCI subjects were instructed to do their best to be close to the frequency and amplitude of the healthy controls. In order to make the ADF more feasible, a small pillow was placed under the participants' popliteal fossa. Undesired motions (knee or hip joint movements) were avoided by putting sandbags on their right knees. During the MI task, all participants were required to imagine that they were performing the same ADF movements as the ME task, without actually executing them. After the experiment, all participants reported that they were able to perform the MI task, and no imagery occurred during their rest blocks.

To ensure the accuracy of their performance throughout the two sessions, each task was first practiced outside and then inside the scan room prior to the scanning procedure. The accuracy of the MI performance was determined by the controllability of the motor imagery (CMI; Naito et al., 2002). Outside the scanner, electromyography (EMG) recordings were done to ensure that all participants had no EMG activity during the MI practice. As EMG is insensitive in detecting small movements caused by gradient-induced artifacts, EMG was not performed inside the scanner. Instead, an operator visually monitored the entire procedure during the tasks. No overt movements were observed during either the MI task or the resting time blocks.

### Post-processing

Post-processing was performed using SPM8 neuroimaging software (http://www.fil.ion.ucl.ac.uk/spm/software/spm8/) and the MATLAB 7.8 platform (Mathworks Inc., Natick, MA, USA). First, the original images were slice-time corrected. Then, they were spatially realigned in order to correct the inter-volume motion displacement. The realignment parameters were checked, and all data were within the lower threshold of 2 mm of translation and 2° of rotation. Next, normalization was performed to register the realigned fMRI data into the standard Montreal Neurological Institute space and convert them into 3 × 3 × 3 mm^3^ voxel sizes. Finally, the normalized data were spatially smoothed using a full-width half-maximum Gaussian kernel of 8 mm^3^.

### Statistical analyses

The preprocessed fMRI data were first subjected to first-level analyses to obtain the individual activation maps of the MI and ME tasks in both groups. Then, a random-effect statistical analysis was performed within each group using the individual activation maps to generate group-wise activation maps. The threshold for significant activation was *q* < 0.01, with a false discovery rate (FDR) method corrected for multiple comparisons at the voxel level and a minimum cluster size of 30 voxels.

A paired *t*-test (with a combined height threshold of a *q* < 0.01 with FDR multiple corrections and a minimum cluster size of 30 voxels) was used to test the differences in activation strengths between the MI and ME tasks, in both the SCI patients and the healthy controls. Conjunction analyses were performed to identify the overlapping ME and MI networks in the ISCI patients and healthy controls (with a height threshold of a *q* < 0.01 with FDR and a minimum cluster size of 30 voxels). Two sample *t*-tests were performed to test the inter-group differences (injury effect) in the activation strengths of both the ME and MI tasks between the ISCI and NC, with age and gender as nuisance covariates (uncorrected voxel-wise *P* < 0.05, with a minimum cluster size of 30 voxels). Finally, Pearson correlation analysis (*P* < 0.05, uncorrected) was performed to determine the association between the duration of the spinal injury and the activation strength of the MI and ME tasks.

To investigate whether the injury level would influence the activation pattern of ME and MI, we further split the ISCI into cervical ISCI and sub-cervical ISCI sub-groups. We used one sample *t*-test to confirm the spatial activation patterns of ME and MI in the cervical ISCI group and sub-cervical ISCI group, separately. Further, conjunction analyses were applied for identifying the similarity in spatial activation patterns between ME and MI of the two ISCI sub-groups, separately, and paired-sample *t*-tests were applied for identifying and distinguishing the spatial activation patterns between ME and MI of the two ISCI sub-groups. Because the sample size for the two group was small (only 6 and 11 patients for sub-cervical ISCI group), we use a relative loose threshold with *p* < 0.01 (uncorrected) and cluster size of 30 voxels. Moreover, a one-way analysis of variance (ANOVA) was performed to test the inter-group differences in the activation strengths of both the ME and MI tasks after extracting the region of interest (ROI) that showed changed activation by voxel-size comparison, with age and gender as nuisance covariates (uncorrected voxel-wise *P* < 0.05, with a minimum cluster size of 30 voxels).

## Results

### The activation patterns of the ME and MI tasks in the NC group

**Figure 1:**
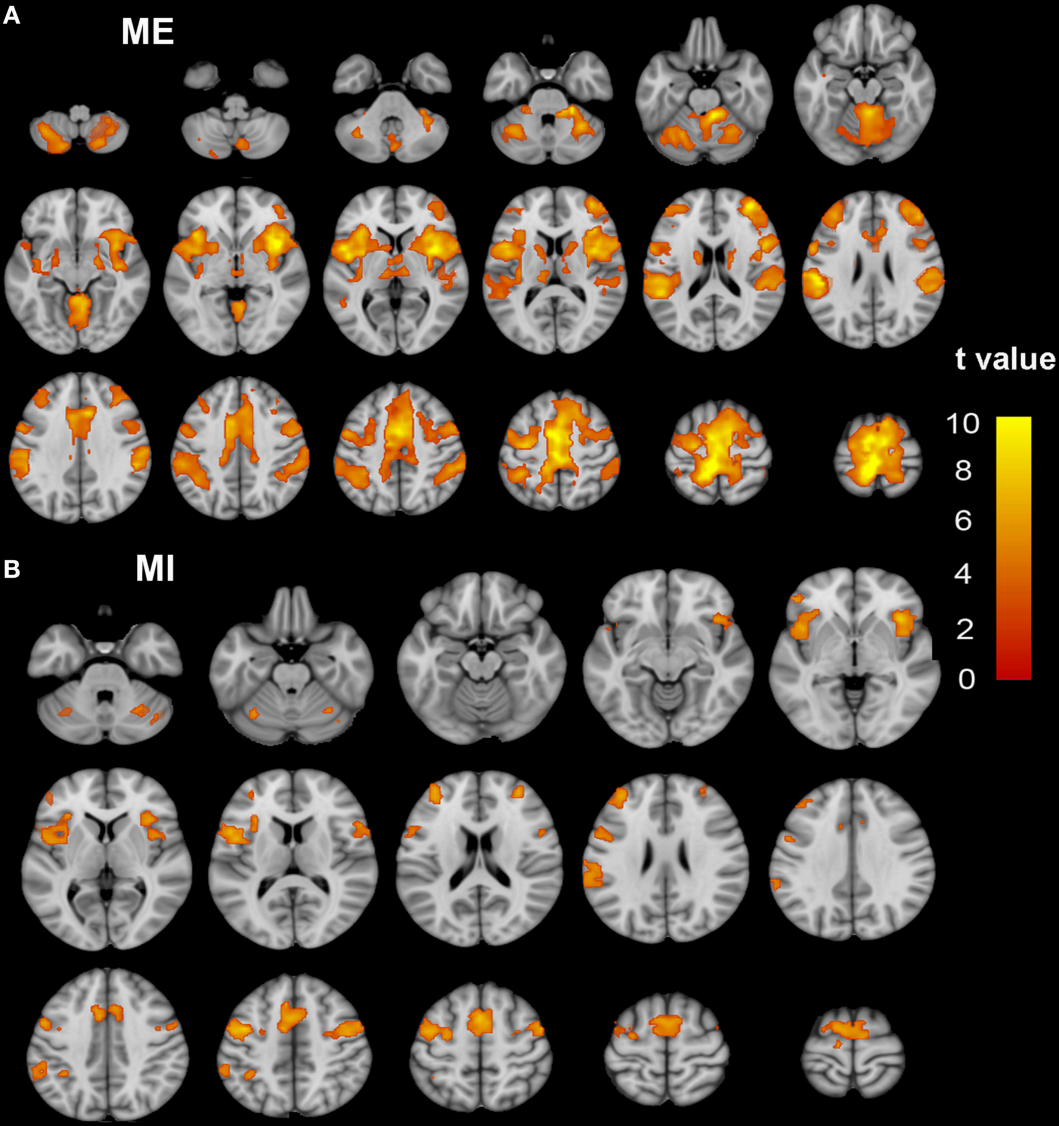
**The activation patterns of the ME and MI tasks in the NC group**. Typical brain regions with activation during an ME task are shown in **(A)**, while brain regions with activation during an MI task are shown in **(B)**. Significant activation is considered to correlate to a voxel-wise threshold of *q* < 0.01 (FDR correction) and a cluster ≥30 voxels. The right side of the image corresponds to the right hemisphere. The color bar represents the *t*-values. ME, motor execution; MI, motor imagery; NC, normal controls.

**Figure 2:**
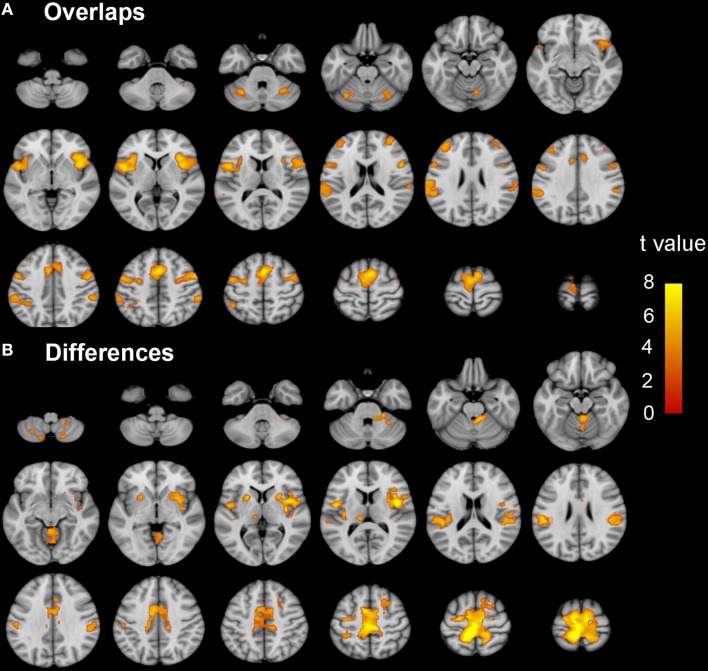
**The coactivation and differences between the MI and ME tasks in the NC group**. Conjunction analyses between the MI and ME tasks shows coactivation in the bilateral SMA, dorsal premotor cortex, ventral premotor cortex, IPL, CB vermal 6, lobules 6, and Crus 1, aINS, dlPFC, and SMG **(A)**. Compared to the ME task, the MI task evoked weaker activation in the bilateral paracentral lobule, SMA, Putamen, SMG, CB lobule 8, right aINS, CB vermal and lobules 4–6, SFG and left thalamus **(B)**. Significant activation was considered at a voxel-wise threshold of *q* < 0.01 (FDR correction) and a cluster ≥30 voxels. The right side of the image corresponds to the right hemisphere. The color bar represents the *t*-values. MI, motor imagery; ME, motor execution; NC, normal controls; SMA, supplementary motor area; IPL, inferior parietal lobule; CB, cerebellum; aINS, anterior insula; dlPFC, dorsolateral prefrontal cortex; SMG, supra marginal gyrus.

Brain regions responding to the ME and MI task in the healthy participants are shown in Figure 1. When a conjunction analysis was performed between the MI and ME tasks, overlapping activation regions are shown in Figure 2A and Table S1. The paired *t*-test was performed to compare differences in the activation amplitudes between the MI and ME tasks in the NC (Figure 2B and Table S1).

### The activation patterns of the ME and MI tasks in the ISCI group

**Figure 3:**
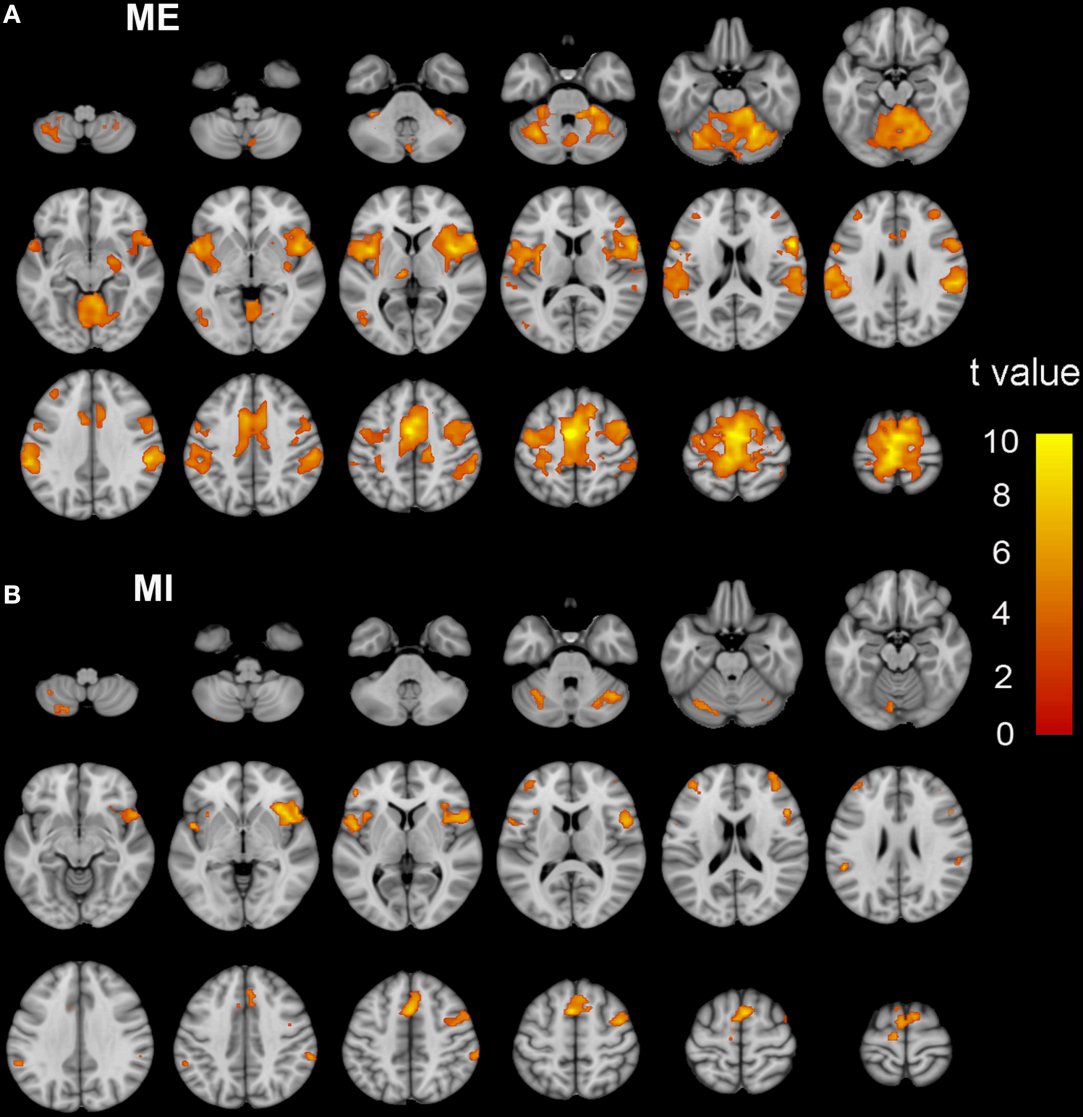
**The activation patterns of the ME and MI tasks in ISCI patients**. Typical brain regions with activation during the ME task are shown in **(A)**, while brain regions with activation during an MI task are shown in **(B)**. Significant activation was considered when the voxel-wise threshold was *q* < 0.01 (FDR correction) and the cluster ≥30 voxels. The right side of the image corresponds to the right hemisphere. The color bar represents the *t*-values. ME, motor execution; MI, motor imagery; ISCI, incomplete spinal cord injury.

**Figure 4:**
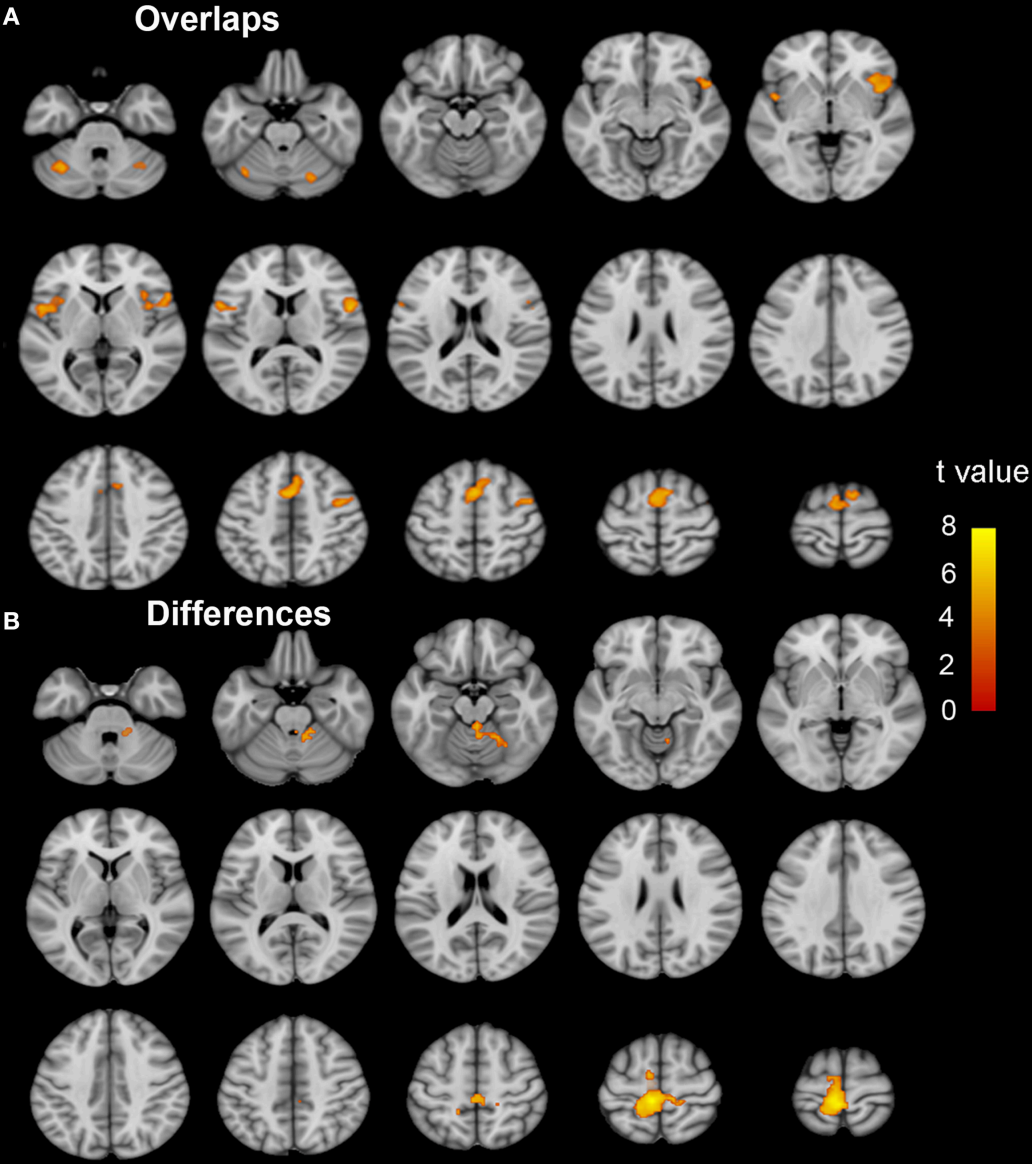
**The coactivation and differences between the MI and ME tasks in ISCI patients**. The coactivation pattern of the MI and ME tasks was similar but weaker than that of the NC group **(A)**. Compared to the ME task, the MI task evoked weaker activation in the bilateral SMA, CB lube 6, IFO, aINS, left CB Crus 1, MFG, left paracentral lobule, precuneus, right CB vermal 4–5 and lobules 4–6 **(B)**. Significant activation was considered at a voxel-wise threshold of *q* < 0.01 (FDR correction) and cluster ≥30 voxels. The right side of the image corresponds to the right hemisphere. The color bar represents the *t*-values. MI, motor imagery; ME, motor execution; ISCI, incomplete spinal cord injury; NC, normal controls; SMA, supplementary motor area; CB, cerebellum; IFO, inferior frontal operculum; aINS, anterior insula; MFG, middle frontal gyrus.

Brain regions responding to the ME and MI task in the ISCI patients are shown in Figure 3. The coactivation pattern of the MI and ME tasks in the ISCI group were also similar to those in the control group, but the distribution of the coactivation pattern was smaller in the ISCI group. In the ISCI group, the coactivation pattern was observed in the following regions: the bilateral supplementary motor area (SMA), the inferior frontal operculum and cerebellum lobule 6, left cerebellum Crus 1, the anterior insula (aINS), and the right middle frontal gyrus (MFG; Figure 4A and Table S2). A paired *t*-test was used to compare the differences in the activation amplitudes between the MI and ME tasks in the ISCI group. Compared with the ME task, the intensity and extent of activation in the aforementioned regions, as well as left paracentral lobule, precuneus and right cerebellum vermal 4–5 and lobules 4–6, were significantly decreased during the MI task (Figure 4B and Table S2).

Brain regions responding to the ME and MI task in cervical ISCI and sub-cervical ISCI sub-groups were shown in Supplementary Figures 1, 2. The coactivation and the difference in the activation patterns between the MI and ME tasks are shown in Supplementary Figure 3 (cervical ISCI group) and Supplementary Figure 4 (sub-cervical ISCI group). Generally the spatial activation patterns of ME and MI were similar between the cervical and sub-cervical ISCI sub-groups, although a relative smaller and noisy distribution in the sub-cervical ISCI group, which may be caused by smaller sample size in this sub-group.

### Differences in activation amplitudes between the ISCI group and the NC group

**Figure 5:**
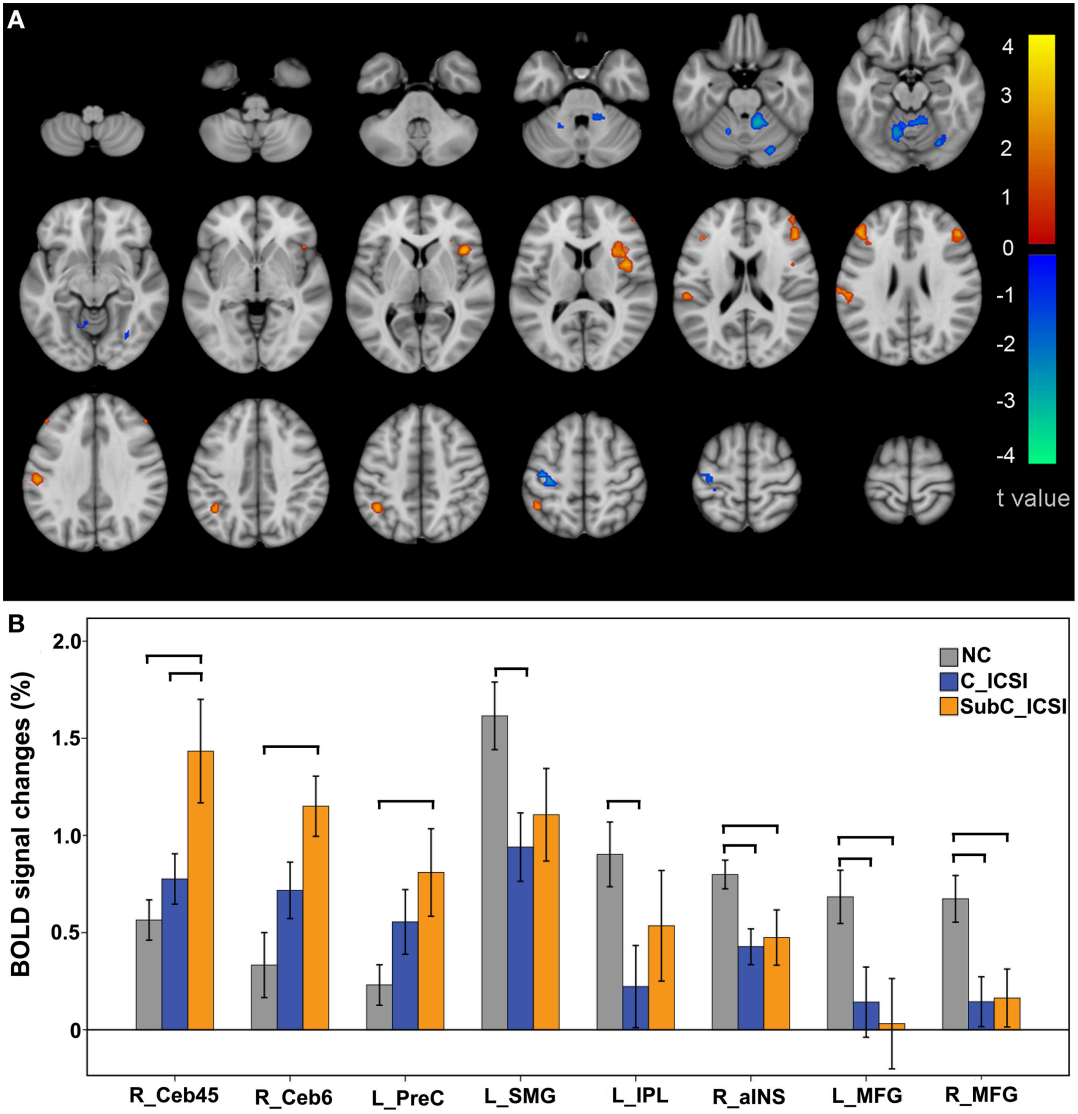
**Intergroup activation differences between the ISCI subjects and NC subjects in the ME task**. Compared to the NC, the ISCI subjects showed increased activation in the left PreCG and right CB and decreased activation in the left SMG, left IPL, right aINS, and bilateral MFG (uncorrected, *P* < 0.05 and cluster ≥30, **A**). Furthermore, the activation increment in the motor-related regions of the cervical ICSI subgroup was not significant compared to the NC subgroup, while the activation decrements in the cognitive-related regions in both the cervical and sub-cervical ISCI sub-groups was comparable to those of the NC subgroup **(B)**. ISCI, incomplete spinal cord injury; NC, normal controls; ME, motor execution; PreCG, precentral gyrus; CB, cerebellum; SMG, supra marginal gyrus; IPL, inferior parietal lobule; aINS, anterior insula; MFG, middle frontal gyrus. The horizontal bar represents a statistically significant difference between the two groups.

During the ME task, increased activation in the motor related regions (such as the left precentral gyrus, the right cerebellum lobe 4–6) and decreased activation in the cognitive-related regions was identified in the ISCI patients compared to the NC group (Figure 5A and Table S3). Furthermore, when we split the ISCI patients into cervical and sub-cervical subgroups, we found that the activation increment in the motor-related regions of the sub-cervical (*P* < 0.05), but not cervical (*P* > 0.05), ICSI subgroup was significant compared to the NC, while the activation decrement in the cognitive-related regions in both the cervical and sub-cervical ISCI subgroups was comparable to the NC (Figure 5B). Pearson correlation analyses did not find any correlation between the activation amplitudes of these areas and the SCI duration (*P* > 0.05).

**Figure 6:**
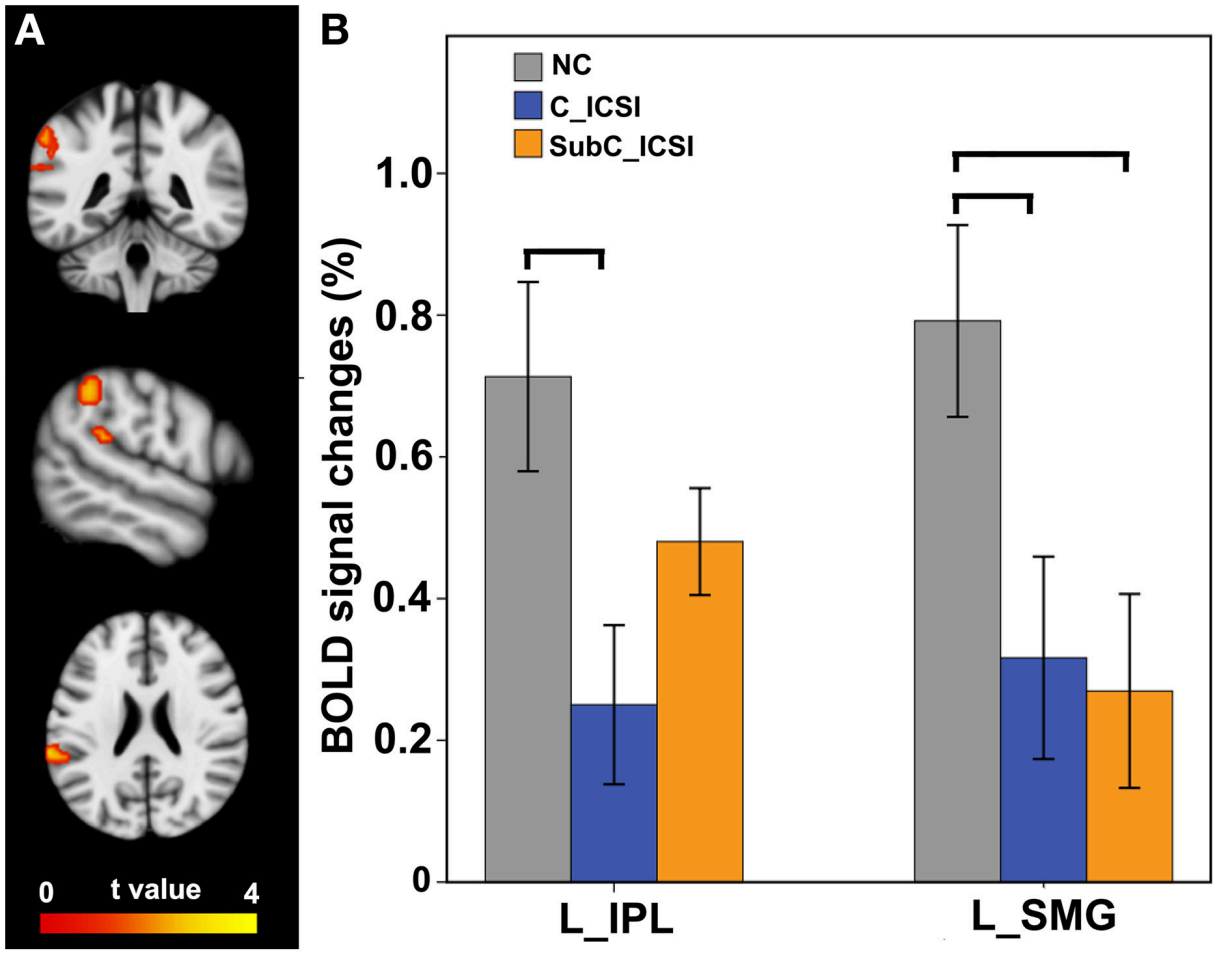
**Intergroup activation differences between the ISCI subjects and the NC subjects in the MI task**. Compared to the NC subjects, the ISCI subjects showed decreased activation in the left SMG and IPL (uncorrected, *P* < 0.05 and cluster ≥30, **A**). Furthermore, the activation decrement in the left IPL of the cervical ICSI subgroup was not significant compared to that of the NC subgroup, while that in the left SMG in both the cervical and sub-cervical ISCI sub-groups was comparable **(B)**. ISCI, incomplete spinal cord injury; NC, normal controls; MI, motor imagery; SMG, supra marginal gyrus; IPL, inferior parietal lobule. The horizontal bar represents a statistically significant difference between the two groups.

During the MI task, subjects with ISCI showed decreased activation in the left SMG and IPL compared to the NC subjects (Figure 6A, and Table S3). Furthermore, the activation decrement in the left IPL of the cervical ICSI subgroup was not significant compared to the NC, while that in the left SMG in both the cervical and sub-cervical ISCI sub-groups was comparable (Figure 6B). Pearson correlation analyses did not find any correlation between the activation amplitudes of these areas and the SCI duration (*P* > 0.05).

## Discussion

The present study explores the functional organization patterns of MI and their changes in ISCI patients. Our findings demonstrate both functional preservation and reorganization during the MI task.

The neural mechanisms of MI are similar to those that occur in the early stages of motor execution (preparation and programming; Garbarini et al., 2013; Hétu et al., 2013; Di Rienzo et al., 2014a), except that the actual movement is voluntarily inhibited during the MI process. In our study, while performing both the MI and ME tasks, similar regions of the brain were activated in both the ISCI subjects and the healthy controls. This indicates that many features of ME and MI activation in healthy subjects were consistent with those in the ISCI subjects. Activating the motor network through MI could help ISCI patients rebuild connections with their affected limb, much like ME therapy. These overlapping regions (the SMA, dorsolateral prefrontal cortex, premotor cortex, IPL, cerebellar lobule VI, and anterior insula) are considered to be involved in motor preparation and execution (Rodriguez et al., 2008). Thus, when establishing a MI training strategy for ISCI patients, the way to mentally strengthen the motor preparation process should be considered.

Both in ISCI patients and healthy controls, brain regions with representation of the right lower limb (left medial precentral gyrus and paracentral lobule) were observed during the ME task but not during the MI task. The involvement of the primary motor cortex during the MI task remains controversial (Hétu et al., 2013). Our results are consistent with those of previous reports (Alkadhi et al., 2005; Lorey et al., 2010). The absence of the primary motor cortex is probably due to the inhibitory influence of SMA (de Lange et al., 2008). According to these findings, MI training may not impact the activity of the primary motor cortex.

We found an increased activation in the motor related regions, including the left precentral gyrus and right cerebellar lobe 4–6, during the ME task. Our result was inconsistent with the results of previous studies, which showed over-activation in the ipsilesional precentral gyrus of chronic sub-cortical stroke patients when they performed motor execution tasks (Tombari et al., 2004; Zhang et al., 2014). Furthermore, the task-related activation of motor related cortices had been reported to be closely associated with motor recovery in the sub-cortical stroke patients, indicating that overactivation of these region is more likely to reflect neuronal reorganization (Ward et al., 2003). We further found that the increased activation of the motor-related regions is mainly due to the sub-cervical ISCI patients, indicating that this over-activation may be due to the reorganization of the impaired corticospinal tract, such that it controls the lower-limb instead of the upper one. It should be noted that the overactivation region in the left precentral gyrus was not within the representation center of the lower limb (as seen Figure 1A) but was more inferior-laterally shifted (roughly near the representation of the upper limb). This shifted activation center has previously been reported in subcortical stroke patients (Calautti et al., 2003; Zhang et al., 2014) and has been shown in monkeys with cortex damage (Nudo et al., 1996). Thus, over-activation of the motor-related regions in the ISCI (especially in sub-cervical patients) suggests that the adjacent intact motor cortex may be reshaped to control the affected lower limb via the remnant corticospinal tract and that the un-deprived motor cortex may play an important role in motor recovery after ISCI (Nudo et al., 1996). For patients with cervical ISCI, the representation cortices for both the lower and upper limbs were (partially) deprived from the peripheral targets. Thus, the “damaged” cortices are harder to reshape than the intact ones. This prediction was supported by a recent study showing different spatial locations between reduced cortical thickness and increased activation in chronic sub-cortical stroke patients (Zhang et al., 2014).

We also found decreased activation in cognitive-related regions during both the ME and MI tasks in the ISCI patients, especially the left IPL and SMG, which are involved in motor attention (Rushworth et al., 2001), motor planning (Ward et al., 2003), and action coding (Fogassi et al., 2005). Furthermore, lesions of the parietal cortex selectively impaired the prediction of hand movement timing, indicating the important roles of these areas in MI (Sirigu et al., 1996). Thus, the decreased activation of the parietal cortex during both ME and MI in the ISCI patients strongly indicates corresponding changes in their mental movement representations. Contrary to our findings, increased activation of the parietal cortex in both the ME and MI tasks has been reported in CSCI patients (Hotz-Boendermaker et al., 2008), indicating that the level of sensory-motor deafferent and deefferent pathways may cause diverse consequences in the parietal activity during mental movement representations. However, the present study does not address the underlying neural mechanisms, which should be clarified in the future.

There were some limitations in our study. First, the main limitations were the heterogeneity (such as the etiology and the injury level) of the included injuries and the relatively small number of patients. Although we found a weak trend of association between the injury level and ME activation in the contralateral precentral gyrus, relatively large ISCI samples with homogenous injury types should be considered to fully clarify the role of the injury level on the functional reorganization of both the MI and ME networks. Second, we used muscle weakness levels to assess the movement impairment of the right ankle, which was too simple to quantify subtle changes in the motor function of the ankles.

In summary, our findings demonstrated that the MI network was functionally preserved in patients with incomplete spinal cord injury. In addition, increased activation in the motor-related regions during the ME task and decreased activation in the parietal regions during both the ME and MI tasks were identified in the ISCI patients compared to the healthy controls, indicating a functional reorganization of these regions after ISCI. The functional preservation and reorganization of the MI network in ISCI patients suggests a potential role of MI training in motor rehabilitation.

## Author contributions

XC: 1. The conception or design of the work; 2. The acquisition, analysis, and the interpretation of data for the work; 3. Drafting the work; 4. Final approval of the version to be published; 5. Agreement to be accountable for all aspects of the work. LW: 1. The conception of the work; 2. The acquisition, analysis data for the work; 3. Final approval of the version to be published; 4. Agreement to be accountable for all aspects of the work. WQ, WZ, ZQ: 1. The analysis data for the work; 2. Drafting the work; 3. Final approval of the version to be published; 4. Agreement to be accountable for all aspects of the work. NC: 1. The design of the work; 2. Revising the work; 3. Final approval of the version to be published; 4. Agreement to be accountable for all aspects of the work. KL: 1. The design of the work; 2. Final approval of the version to be published; 3. Agreement to be accountable for all aspects of the work.

## Funding

The National Science Foundation of China (No. 81271556). Beijing Municipal Administration of Hospitals Clinical Medicine Development of Special Funding Support (ZYLX201609). Key Projects in the National Science & Technology Pillar Program during the Twelfth Five-Year Plan Period (2012BAI10B04). Beijing Municipal Natural Science Foundation (No. 7113155). The Science Foundation of Beijing municipal commission of education (No. KM201210025013).

### Conflict of interest statement

The authors declare that the research was conducted in the absence of any commercial or financial relationships that could be construed as a potential conflict of interest.

## Abbreviations

ADF: ankle dorsi-plantar flexion
aINS: anterior insula
CSCI: complete spinal cord injury
IFO: inferior frontal operculum
ISCI: incomplete spinal cord injury
IPL: inferior parietal lobule
ME: motor execution
MFG: middle frontal gyrus
MI: motor imagery
NC: normal control
PPC: posterior parietal cortex
SMA: supplementary motor area
SMG: supra marginal gyrus.
