# Supplementary material for: Functional Preservation and Reorganization of Brain during Motor Imagery in Patients with Incomplete Spinal Cord Injury: A Pilot fMRI Study
Source: Front Hum Neurosci. 2016 Feb 15;10:46. doi: 10.3389/fnhum.2016.00046 (PMC4753296; doi:10.3389/fnhum.2016.00046)
Supplement: Supplementary file 1 [file Table1.DOCX]

**Table S1. Comparison of the activation strength between the ME and MI tasks in the NC group.**

| **Cluster** | **Region(AAL)** | **Coordinates** | | |  |  |
| --- | --- | --- | --- | --- | --- | --- |
|  |  | **X** | **Y** | **Z** | **cluster** | **T value** |
|  | **Co-activation** |  |  |  |  |  |
| 1 | SMA_B | 0 | 12 | 51 | 823 | 8.52 |
| 2 | aINS _R, IFO_R | 42 | 12 | 0 | 780 | 8.33 |
| 3 | aINS_L, IFO_L | -48 | 6 | 6 | 689 | 8.19 |
| 4 | CB6_L, CBCrus1_L | -30 | -63 | -27 | 96 | 6.58 |
| 5 | MFG_L | -36 | 45 | 24 | 152 | 5.83 |
| 6 | CB6_R, CBCrus1_R | 33 | -57 | -27 | 121 | 5.69 |
| 7 | SMG_L ,IPL_L | -63 | -21 | 27 | 457 | 5.68 |
| 8 | SMG_R | 60 | -36 | 45 | 152 | 5.53 |
| 9 | MFG_R | 36 | 51 | 18 | 82 | 5.33 |
|  | **Difference** |  |  |  |  |  |
| 1 | PRL_L | -6 | -39 | 63 | 2286 | 11.63 |
|  | PCL_B | -3 | -27 | 66 | 2286 | 9.77 |
|  | SMA_B | -9 | -9 | 72 | 2286 | 9.41 |
| 2 | PUT_R, RO_R, aINS_R | 45 | 3 | 9 | 458 | 9.35 |
| 3 | STG_L | -45 | -33 | 21 | 291 | 8.47 |
|  | RO_L | -36 | -30 | 21 | 291 | 5.97 |
|  | SMG_L | -51 | -27 | 27 | 291 | 6.99 |
| 4 | Vermis45,CB3456_R | 12 | -39 | -27 | 303 | 8.31 |
| 5 | RO_L , aINS_L | -45 | -3 | 12 | 85 | 7.73 |
| 6 | CB8_R | 15 | -54 | -57 | 74 | 7.4 |
| 7 | PUT_L | -21 | 9 | 3 | 48 | 7.22 |
| 8 | SMG_R | 48 | -30 | 21 | 224 | 6.55 |
| 9 | Th_L | -9 | -27 | 12 | 37 | 5.48 |
| 10 | CB8_L | -9 | -75 | -51 | 39 | 5.22 |

Note: All brain voxels are signiﬁcant at a threshold of voxel-wise q＜0.01 (FDR correction) and a cluster size ≥30 voxels. AAL = anatomic automatic labeling; aINS= anterior insula; B = bilateral; CB = cerebellum; IFO = inferior frontal operculum; IPL = inferior parietal lobule; L = left; MFG = middle frontal gyrus; PCL = paracentral lobule; PRL = precuneus lobe; PUT = putamen; R = right; RO = Rolandic operculum; SMA = supplementary motor area; SMG = supra marginal gyrus; Th = Thalamus.
