# Supplementary material for: Functional Preservation and Reorganization of Brain during Motor Imagery in Patients with Incomplete Spinal Cord Injury: A Pilot fMRI Study
Source: Front Hum Neurosci. 2016 Feb 15;10:46. doi: 10.3389/fnhum.2016.00046 (PMC4753296; doi:10.3389/fnhum.2016.00046)
Supplement: Supplementary file 2 [file Table2.DOCX]

**Table S2.** Comparison of the activation strength between the ME and MI tasks in the ISCI group.

| **Cluster** | **Region(AAL)** | **Coordinates** | | |  |  |
| --- | --- | --- | --- | --- | --- | --- |
|  |  | **X** | **Y** | **Z** | **cluster** | **T value** |
|  | **Co-activation** |  |  |  |  |  |
| 1 | MFG_R | 39 | 0 | 51 | 57 | 5.16 |
| 2 | CB6_R | 36 | -60 | -27 | 34 | 5.32 |
| 3 | IFO_L, aINS_L | -48 | 6 | 6 | 109 | 5.83 |
| 4 | CBCrus1_L, CB_6_L | -30 | -60 | -30 | 57 | 5.87 |
| 5 | aINS_R, IFO_R | 54 | 6 | 12 | 223 | 6 |
| 6 | SMA_L, SMA_R | -3 | 9 | 51 | 318 | 6.42 |
|  | **Difference** |  |  |  |  |  |
| 1 | PCL_L,PRL_L | -6 | -33 | 63 | 405 | 9.42 |
| 2 | CB456_R | 3 | -39 | -21 | 92 | 6.38 |

Note: All brain voxels are signiﬁcant at a threshold of voxel-wise q＜0.01 (FDR correction) and a cluster size ≥30 voxels. AAL = anatomic automatic labeling; aINS= anterior insula; CB = cerebellum; IFO = inferior frontal operculum; L = left; MFG = middle frontal gyrus; PCL = paracentral lobule; PRL = precuneus lobe; R = right; SMA = supplementary motor area.
