# Supplementary material for: Functional Preservation and Reorganization of Brain during Motor Imagery in Patients with Incomplete Spinal Cord Injury: A Pilot fMRI Study
Source: Front Hum Neurosci. 2016 Feb 15;10:46. doi: 10.3389/fnhum.2016.00046 (PMC4753296; doi:10.3389/fnhum.2016.00046)
Supplement: Supplementary file 3 [file Table3.DOCX]

**Table S3.** Intergroup differences in the activation strength during the ME and MI tasks

|  |  | **Coordinates** | | |  |  |
| --- | --- | --- | --- | --- | --- | --- |
| **Cluster** | **Region(AAL)** | **X** | **Y** | **Z** | **size** | **T value** |
| **ME** |  |  |  |  |  |  |
| 1 | MFG_R | 48 | 33 | 21 | 50 | 3.24 |
| 2 | aINS_R,IFO_R | 42 | 18 | 6 | 91 | 3.18 |
| 3 | IFT_L, MFG_L | -45 | 33 | 24 | 35 | 2.83 |
| 4 | IPL _L | -42 | -51 | 48 | 43 | 2.57 |
| 5 | SMG_L | -54 | -21 | 33 | 50 | 2.44 |
| 6 | PreCG_L | -33 | -24 | 54 | 35 | -2.76 |
| 7 | CB_6_R | 30 | -69 | -15 | 33 | -2.83 |
| 8 | CB_4_5_R | 15 | -45 | -27 | 127 | -2.89 |
|  | CB_6_L | -15 | -60 | -18 | 127 | -2.43 |
| **MI** |  |  |  |  |  |  |
| 1 | SMG_L | -66 | -36 | 21 | 30 | 3.19 |
| 2 | IPL_L | -51 | -45 | 36 | 30 | 2.69 |

Note: All brain voxels are signiﬁcant at a threshold of voxel-wise q＜0.01 (FDR correction) and a cluster size of ≥30 voxels. AAL=anatomic automatic labeling; aINS = anterior insula; CB = cerebellum; IFO = inferior frontal operculum; IFT = Inferior frontal triangle; IPL = inferior parietal lobule; L = left; MFG = middle frontal gyrus; PreCG = precentral gyrus; R = right; SMA = supplementary motor area; SMG = supra marginal gyrus.
