# Supplementary material for: Functional Preservation and Reorganization of Brain during Motor Imagery in Patients with Incomplete Spinal Cord Injury: A Pilot fMRI Study
Source: Front Hum Neurosci. 2016 Feb 15;10:46. doi: 10.3389/fnhum.2016.00046 (PMC4753296; doi:10.3389/fnhum.2016.00046)
Supplement: Supplementary file 4 [file Image1.PDF]

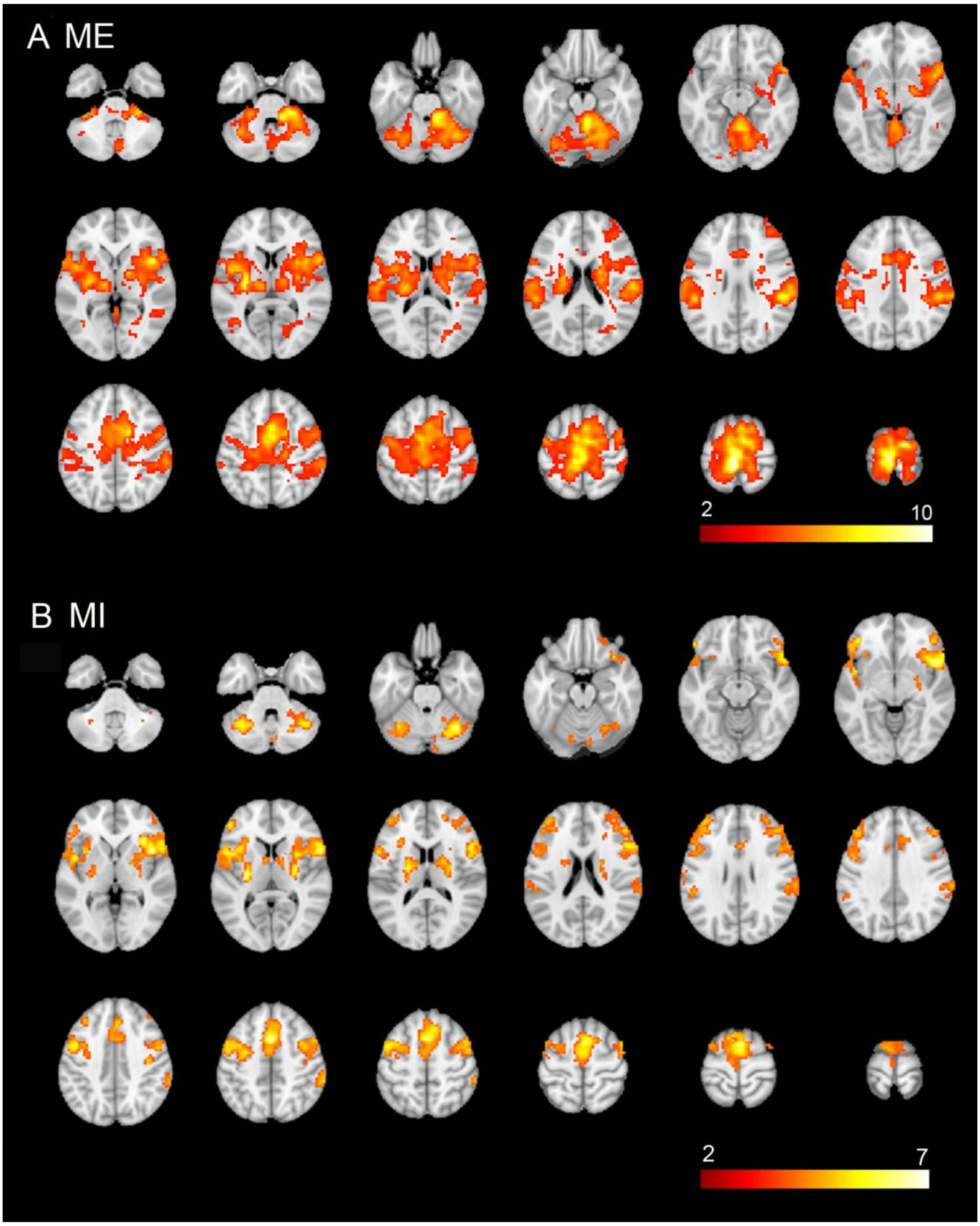

Supplementary Figure 1. The activation patterns of the ME and MI tasks in the cervical ISCI group. Typical brain regions with activation during the ME task are shown in panel A, while brain regions with activation during an MI task are shown in panel B. Significant activation was considered when the voxel-wise threshold was $p < 0.01$ (uncorrected) and the cluster ≥30 voxels. The right side of the image corresponds to the right hemisphere. The color bar represents the t-values. ME = motor execution; MI = motor imagery; ISCI = incomplete spinal cord injury.
