# Supplementary material for: Functional Preservation and Reorganization of Brain during Motor Imagery in Patients with Incomplete Spinal Cord Injury: A Pilot fMRI Study
Source: Front Hum Neurosci. 2016 Feb 15;10:46. doi: 10.3389/fnhum.2016.00046 (PMC4753296; doi:10.3389/fnhum.2016.00046)
Supplement: Supplementary file 7 [file Image4.PDF]

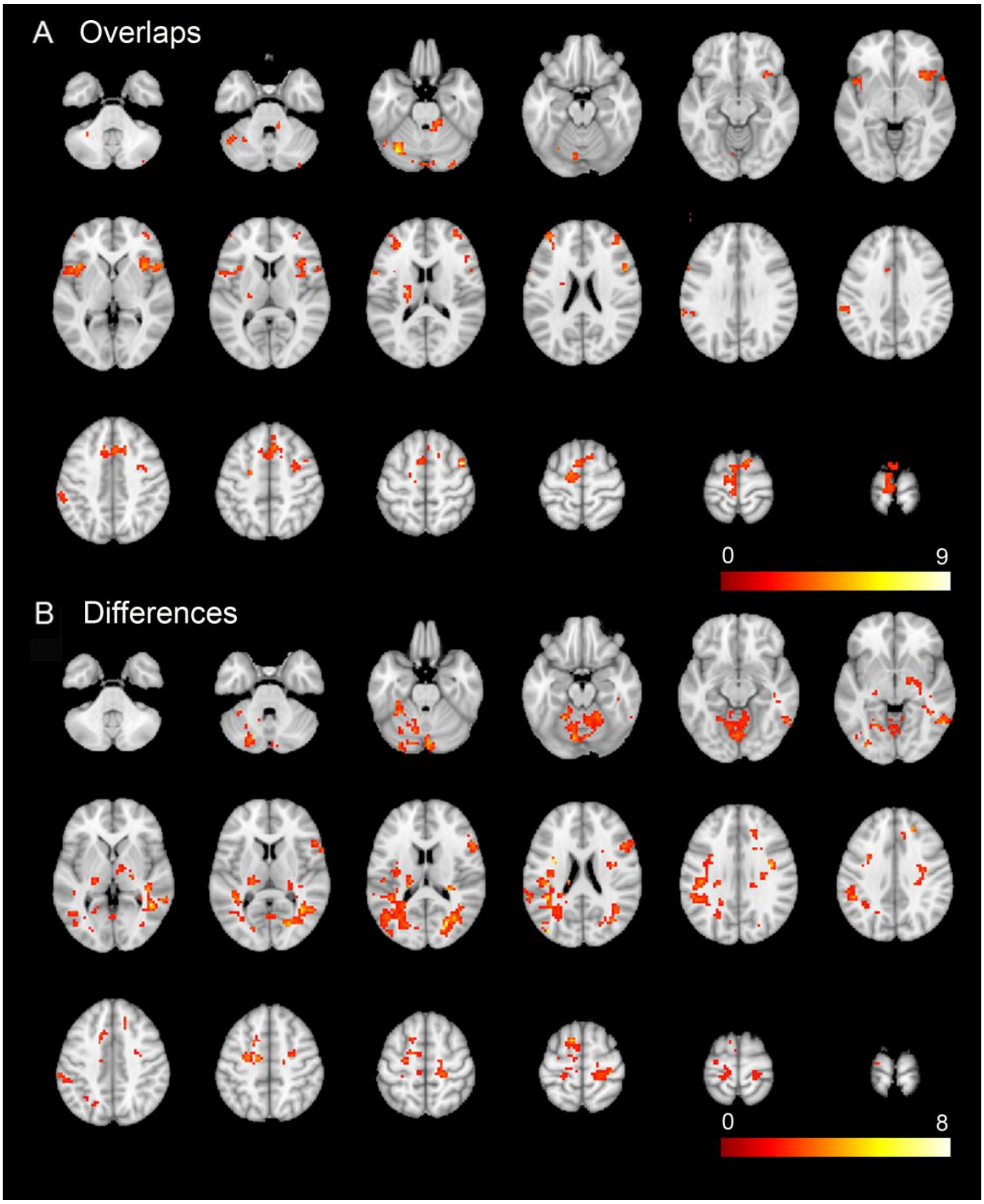

Supplementary Figure 4. The coactivation and differences between the MI and ME tasks in the sub-cervical ISCI group. Significant activation was considered at a voxel-wise threshold of $p < 0.01$ (uncorrected) and cluster ≥30 voxels. The right side of the image corresponds to the right hemisphere. The color bar represents the t-values.
